# Consumer Acceptance of Biscuits Supplemented with a Sorghum–Insect Meal

**DOI:** 10.3390/nu12040895

**Published:** 2020-03-25

**Authors:** Temitope D. Awobusuyi, Kirthee Pillay, Muthulisi Siwela

**Affiliations:** Department of Dietetics and Human Nutrition, University of KwaZulu-Natal, Pietermaritzburg 3209, South Africa; temmybusuyi@gmail.com (T.D.A.); pillayk@ukzn.ac.za (K.P.)

**Keywords:** insects, entomophagy, sorghum, biscuits, consumer acceptance

## Abstract

Insects are abundant in the predominantly sub-Saharan Africa region and are generally high in protein. Wheat grain contains gluten that is vital for the quality of baked goods but does not grow well in warm regions. Partial substitution of wheat with sorghum and insect in biscuits could contribute to food security among vulnerable populations. This study identified insect types most commonly consumed by the rural Olugboja community living in the rural part of the Ikare-Akoko local government area of Ondo State, Nigeria and consumer acceptance of biscuits supplemented with a sorghum and insect meal. Whole grain sorghum meal and insect meal were blended at a ratio of 3:1 (w/w sorghum: insect). Composite biscuits were made by partially substituting wheat flour with the sorghum–insect meal at 20%, 40%, and 60% (w/w). Wheat biscuit (100%) was used as a control. Regular consumers of biscuits (*n* = 84) evaluated the acceptability of the biscuit samples using a five-point facial hedonic scale, which was followed by focus group discussions (FGDs) to assess consumer perceptions of the use of insect as a food source. Biscuits containing the sorghum–insect meal (mean = 4.0 ± 0.6) were more acceptable than the control (3.58 ± 0.6). The biscuits supplemented with 20% of the sorghum–insect meal were the most acceptable (mean = 4.23 ± 0.6) compared to those with higher concentrations (40% and 60%). FGDs revealed that the taste of the biscuits was an important motivation for consumers to accept insect as a food source.

## 1. Introduction

Globally, food insecurity and undernutrition have been found to be prevalent amongst the rural and poor communities. It is a complex issue that characterizes the current world economy [1]. According to the Food and Agriculture Organization of the United Nations [2], food insecurity and hunger have been increasing throughout the world, especially in developing countries. This problem is further compounded by rapid population growth as the global population is estimated to reach 9 billion people by 2050 and this will demand increased output from available agro-ecosystems [3]. Greater pressure on the environment, agricultural land, water resources, fish and meat supply, biodiversity, as well as an increased need for nutrients is predicted [4]. This indicates that there is an urgent need for the reassessment of some unconventional approaches to obtain nutrients, especially protein [5]. One of the many ways of contributing to addressing food security is the consumption of insects [3]. This is because insects are widely abundant, globally, they reproduce quickly, have high growth and feed conversion rates, and a low environmental footprint over their entire life cycle [3].

Edible insects have also gained much attention for their high nutritional value, especially their high protein content [6]. Insects can be processed into food relatively easily. Some species can be consumed whole and can also be processed into pastes or ground into meal, and their proteins can be extracted [3]. The consumption of insects by humans is a well-established, although a diminishing practice in many parts of the world [7]. The practice seems to be culturally universal, only varying with location, type of insect, and the ethnic group involved [8]. About 1900 insect species are eaten worldwide, of which 524 are consumed mainly in developing countries [9]. Edible insect species mainly belong in the traditional and informal food systems [10]. Many cultural and psychological barriers hinder consumer acceptance of insects as a food source [11,12]. Whether insects are eaten or not often depends on customs, preferences, and prohibitions [13], and among some population groups it is just taboo [11]. One of the barriers to the use of insects as a viable protein food source is the low consumer acceptance of insects [14]. Although a large variety of insects are valued as tasty delicacies in many cultures around the world [15,16], the vast majority of consumers react negatively to the prospect of ingesting creatures that are more familiar as pests than as food [12]. Ethical arguments have proven ineffective in persuading consumers to fully accept more sustainable meat alternatives [17]. Consumers are generally unwilling to sacrifice the pleasures they derive from meat for uncertain and delayed benefits [18,19]. While these arguments have contributed to an increased interest in insects as a food source, few consumers are actually ready to adopt insects as part of their regular diet [12,20]. The poor acceptance of insects has often been attributed to their low sensory appeal and unfamiliarity with insects as food [20,21,22,23]. Perhaps, the unappealing aspect of entomophagy is the consumption of the whole insect [24]. People may consume insects more easily if unrecognizable (milled) and incorporated into food. Such a strategy is simple and could overcome the documented low consumer acceptance of whole insects [24].

Sorghum, an indigenous African cereal, is unique because it is a drought-tolerant staple food for over 500 million people living in the arid and semi-arid tropics where maize cannot grow [25,26]. Cereals such as sorghum and wheat, which are principal sources of food in most African households, are therefore the most suitable vehicles for delivering essential nutrients such as protein to vulnerable groups [27]. However, the non-availability and high cost of wheat creates a need to substitute wheat flour with other cereal flours such as sorghum, which is cheaper, accessible, and more sustainable [28]. Sorghum grain is, however, low in protein content, while lysine, threonine, and tryptophan are all limiting amino acids in sorghum [29].

Biscuits are a form of confectionery with a very low water activity and increased shelf-life [30]. They are baked products that are consumed extensively all over the world as a snack food item by both children and adults and on a large scale in developing countries [30]. They can also serve as a source of important nutrients if made available to the population [30]. With the aforementioned nutritional benefits of insects and the appealing nature of biscuits, it may be beneficial to incorporate sorghum and insects into a ready-to-eat snack, such as biscuits, to address the issue of food insecurity in developing countries.

Quite a number of researchers have written widely on entomophagy among several tribes in Nigeria [31,32,33,34,35], but the consumption level is not nutritionally significant. Further, most researchers maintain that, although the practice is more prevalent among rural populations, prejudice against entomophagy still exists within some population groups. On the other hand, the common methods of preparing insects used by rural communities in Nigeria are boiling and frying. Incorporating insects into popular value-added baked products, such as biscuits, could promote their utilization as a food source. In addition, the advantages of biscuits relative to other food products have been stated above. Therefore, this study aimed to determine the consumer acceptance of biscuits supplemented with sorghum and insects through sensory evaluation and focus group discussions, by the rural community of Olugboja in Ondo state, Nigeria.

## 2. Materials and Methods

### 2.1. Preparation of Sorghum Meal

Sorghum grains were purchased from the main market in Ondo State, Nigeria. The sorghum grains were cleaned to remove dirt, stones, and other extraneous materials. The cleaned grains were milled using a laboratory hammer mill fitted with a 0.4 mm screen. The sorghum meal obtained was partially substituted with wheat flour at varying proportions (Table 1). The ratio 3:1 (sorghum: insect) was chosen after preliminary trials in the laboratory revealed that other substitution levels of sorghum and insect resulted in biscuits that were too brittle.

### 2.2. Determination of the Most Commonly Consumed Insect Types

Regular consumers of insects in rural Olugboja community were approached and informed about the details of the study and recruited based on their interest to participate. The most commonly consumed insects were determined using a survey questionnaire. The questionnaire was designed in English and *Yoruba* (local language) for participants who were not proficient in English. Insect consumers (*n* = 79) between the ages of 28 to 56 years (68%; *n* = 54 female and 32%; *n* = 25 male) participated in the study. The questionnaire showing pictorial representation of four commonly consumed insects (palm weevil, termites, grasshoppers, and crickets) was distributed to all participants. The options of commonly consumed insects were selected based on their availability in the study area. The participants were asked to rank the insects in order of most consumed/preferred to the least consumed, where 1 was the most consumed/preferred insect and 4 was the least consumed/preferred insect. The most preferred insect of the four was used together with sorghum to supplement the biscuits used in the consumer acceptability studies.

### 2.3. Preparation of Insect Meal

Termite, was the most preferred insect in rural Olugboja community as determined by the survey outlined in Section 2.2. The insect, purchased from the market was washed, oven dried, and milled into a meal with a laboratory mill. The resulting insect meal was mixed with the sorghum meal and wheat flour at different proportions in the biscuit formulation as shown in Table 1.

### 2.4. Biscuit Preparation

**Figure 1:**
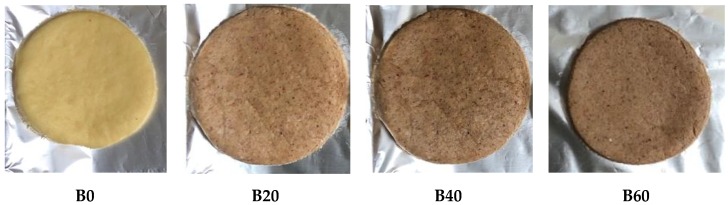
Biscuits before baking. **B0**: Control (100% wheat); **B20**: 15% sorghum and 5% insect substitution level; **B40**: 30% sorghum and 10% insect substitution level; **B60**: 45% sorghum and 15% insect substitution level.

**Figure 2:**
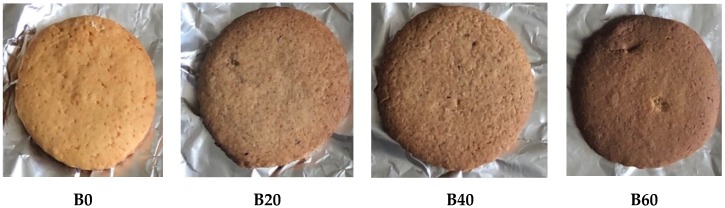
Biscuits after baking. **B0**: Control (100% wheat); **B20**: 15% sorghum and 5% insect substitution level; **B40**: 30% sorghum and 10% insect substitution level; **B60**: 45% sorghum and 15% insect substitution level.

Control biscuits were prepared according to the method described by De Jager [36]. Dry ingredients: flour (480 g), sugar (200 g), powdered milk (50 g), vanilla essence (5 mL), salt (5 mL), and baking powder (10 mL) were sieved into a mixing bowl and mixed together for 3–5 min. Margarine (250 g) was added and the mixture was kneaded for 2 min to form a firm dough. The dough was rolled out on a tray using a rolling pin and cut into desired shapes using a biscuit cutter. The dough pieces were then transferred onto a baking sheet lined with aluminium foil (Figure 1). Biscuits were baked in a preheated oven at 150 °C for 20 min and thereafter cooled for 30 min at room temperature (Figure 2). Biscuits supplemented with the sorghum–insect meal were prepared using the above method, but with different substitutions of sorghum and insect (ratio 3:1). Wheat flour was substituted with 15% sorghum meal and 5% insect meal to produce the biscuit sample of 20% substitution (B20). Sample B40 contained 30% sorghum meal and 10% insect meal, whilst B60 contained 45% sorghum meal and 15% insect meal (see Table 1). The control biscuit (B0) had neither sorghum nor insect added to it.

### 2.5. Sensory Evaluation

#### 2.5.1. Questionnaire Development

A consent form in English and *Yoruba* (local language) and a sensory evaluation questionnaire developed both in English and *Yoruba*, were used in this study. The questionnaire made use of a five-point facial hedonic scale (1 = very bad; 5 = very good), and assessed the following attributes: taste, texture, aroma, color, and overall acceptability. The facial hedonic scale is a popular sensory evaluation tool that indicates the degree of likeness and is also more suitable for people with low literacy [37].

#### 2.5.2. Recruitment of Panelists

The recruitment of panelists was done 48 h before the sensory evaluation data collection. About 120 people, both adult male and female living in rural Olugboja community were verbally approached in their homes for inclusion in the study. They were asked if they consumed insects and biscuits and whether they had any allergies. Those who consumed biscuits and insects, or were willing to try, and had no allergies, were included in the study. This was done to determine the acceptability of insect as a biscuit ingredient among biscuit and insect consumers. The number of panelists to be enrolled in a hedonic test should range from 50 to 100 [38]. Given this, a simple random approach was employed and a Table of Random Numbers [39] was used to decide which of the 120 volunteers would be included. Each of the 120 participants was assigned a number from 1 to 120. Using the Table of Random Numbers, the last two digits of the numbers listed on the chart were used in the selection process. In the process of selection, if the first number to match the numbers allocated to a participant was 896, this meant that the participant with the number 89 (first two digits) would be included in the study. If any of the participants did not have that number, the next number in line or in the column was chosen. About 100 participants were selected in all, however, only 84 participants were available for the sensory evaluation. This led to the recruitment of 84 panelists between the ages of 20 and 59 years. The selected panelists were then informed about the purpose, procedures and importance of the study. They were further asked to sign a consent form either in English or *Yoruba* (local language) before the commencement of the sensory evaluation.

#### 2.5.3. Orientation of Panelists

A 15-min orientation session was conducted in a hall situated in the rural Olugboja community before the sensory evaluation commenced. The session was conducted using both English and *Yoruba* (local) languages. The purpose of the orientation was to explain to the panelists the importance of the study. It was also emphasized that panelists should not communicate with one another during the evaluation so as not to influence each other. Sensory attributes including aroma, texture, taste, color, and overall acceptability were explained to the panelists. The facial hedonic scale on the questionnaire was explained as well as the procedure for evaluating the biscuits. Water was provided to cleanse the palate before and in between tasting the samples. The panelists were also informed that they could withdraw from the study at any point if they wanted to, without any negative consequences. After the orientation was complete, panelists were given a 15-min break before commencement of the sensory evaluation. Research assistants were present to help participants where necessary.

#### 2.5.4. Procedure for Data Collection

Evaluation of biscuits supplemented with the sorghum–insect meal was carried out over a five-hour period, 15-min after the orientation of panelists and 48 h after recruitment of panelists. The sensory evaluation groups consisted of 16–18 people at a time and a total of 84 panelists evaluated the samples over the five-hour period. This was done to ensure that the hall was not overcrowded, and panelists were individually seated so as not to influence each other. The reason for the seating arrangement was explained to the panelists prior to the evaluation. During the sensory evaluation, panelists were presented with the biscuit samples on a polystyrene plate. Three-digit random codes were determined using a Table of Random Permutations of Nine [39] to identify the samples. The three-digit random codes were placed on removable labels. The identity of the samples was known to the researcher, but not the panelists. This was done so that the responses of the person sitting next to them would not influence their own evaluation. A sensory evaluation questionnaire with the five-point facial hedonic scale (1 = very bad; 5 = very good), developed in both English and *Yoruba* (local language) was distributed to panelists. The panelists thereafter evaluated the biscuit samples based on the following sensory attributes: aroma, texture, taste, color, and overall acceptability. Panelists wrote the three-digit code allocated to a specific biscuit sample on the questionnaire, before the sensory evaluation commenced. This was done to ensure that the evaluation given correlated with the correct biscuit sample. Panelists were provided with two whole biscuits of each biscuit sample to be evaluated. Each panellist received the biscuits in a random serving order, pre-determined using a Table of Random Permutations of Nine. After completing the first round of evaluations, the left-over samples were collected and discarded, and the panelists commenced the evaluation of a new set of biscuits. The same procedure was repeated until all biscuit samples had been evaluated.

#### 2.5.5. Focus Group Discussions

Focus group discussions (FGDs) were conducted with some of the panelists after the sensory evaluation to determine their perceptions of the use of insects as a food source. Thirty sensory evaluation participants volunteered to participate in the FGDs. Self-selection of FGDs panelists was in accordance with research ethics of voluntary participation. It was assumed that self-selection would not have an influence on the outcome of the discussions. There was a total of four focus group discussion sessions conducted with varying numbers of participants per group. The number of participants ranged from four to seven per group. The ideal size for a focus group discussion for most topics is five to eight participants [40]. Focus group discussion questions were generated prior to the study in English and translated into *Yoruba* (local language). The focus group discussions were also held in both English and *Yoruba* languages. The focus group questions were validated by the study supervisors who checked that the questions met the objectives of the FGDs. The FGD questions were tested on a group of people living in rural Olugboja community, who share similar demographic characteristics to the study participants, during a pilot study. Participants gave consent to participate in the discussion and for the discussion to be voice recorded by signing consent forms in English and *Yoruba*, before the FGDs commenced. The FGDs were facilitated by the researcher who was fluent in English and *Yoruba* and had experience working with FGDs. Discussions were recorded using a voice recorder. The recordings were later transcribed, and the transcripts were translated into English verbatim by the researcher and checked for accuracy by the research assistant, who was also fluent in English and *Yoruba* (local language).

#### 2.5.6. Ethics Approval

This study was ethically reviewed and approved by the University of KwaZulu-Natal, Biomedical Research Ethics Committee (Reference number: BE265/18). The Akoko North-East Local Government Authority also issued a supporting letter for the research to be conducted.

#### 2.5.7. Statistical Analysis

The Statistical Package for Social Sciences (SPSS) version 20 was used to analyse the data. Mean acceptability scores were computed. One-way analysis of variance (ANOVA) was performed and the means compared using Fisher’s Least Significant Difference (LSD) test (*p* < 0.05).

## 3. Results

### 3.1. Most Commonly Consumed Insect Types

**Figure 3:**
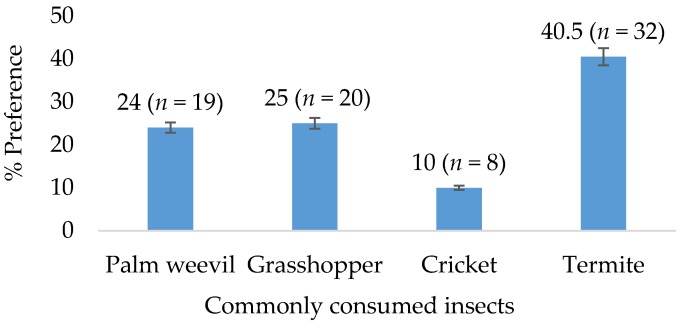
Survey results for the most commonly consumed insects (*n* = 79).

A total of 79 survey questionnaires showing four different species of insects were completed by regular consumers of insects. The result showed that the most preferred insect in the rural Olugboja community was termite (40.5%; *n* = 32) (Figure 3). This was followed by grasshopper (25%; *n* = 20), palm weevil (24%; *n* = 19), and the least preferred was cricket (10%; *n* = 8). Termite was then used in baking biscuits that were evaluated for sensory attributes.

### 3.2. Consumer Acceptability of Biscuits Supplemented with Sorghum–Insect Meal

The number and age of panelists who participated in the sensory evaluation are presented in Table 2. The consumer acceptance evaluation was carried out using 84 panelists. Among the panelists, 48.8% (*n* = 41) were male, while 51.2% (*n* = 43) were female. About 65.5% (*n* = 55) of the panelists were between the ages of 20–39 years, while 34.5% (*n* = 29) were above 40 years of age (Table 2). The color of biscuit samples supplemented with the sorghum–insect meal scored higher compared to the control (100% wheat biscuits) (Table 3). The intense brown color in biscuits was observed with an increase in insect concentration and at 15% substitution level (B60), the color was the least acceptable to consumers. The results could be attributed to the fact that the insect meal was brown, whilst the sorghum flour was cream in color. Biscuits supplemented with 5% insect substitution level (B20) was rated higher for taste than those with higher concentrations of insects (B40 and B60, respectively). Additionally, biscuits supplemented with the sorghum–insect meal had slightly higher acceptability scores for aroma, when compared to the control. The texture of biscuit samples containing insect was liked and rated the same as that of the control (100% wheat biscuits).

### 3.3. Focus Group Discussions

The demographic characteristics of panelists who participated in the focus group discussions is presented in Table 4. Focus group discussions were attended by 30 participants who had earlier taken part in the sensory evaluation. The majority were female (70%; *n* = 21), with 30% (*n* = 9) male. Perceptions of the participating consumers regarding the use of insects as a food source are presented in Table 5.

## 4. Discussions

### 4.1. Most Commonly Consumed Insect Types

Participants who participated in the survey revealed that among the insects, termite was the most preferred because it is locally available, cheap, takes less time to cook, and also tastes better when compared to other insects. Palm weevil was said to be too chewy and takes longer to cook. Participants mentioned that preparing and processing of grasshopper can become tedious as it involves removing the wings, heads, and other parts before consumption, although it is locally available. Cricket was the least liked because it takes a longer time to cook, is very expensive, and is bland. During the survey, some participants, however, expressed their ignorance when asked about the potential health benefits of insects. Although the participants had been buying and consuming insects for a long time, they did not know much about their health benefits. This shows that although entomophagy is a common practice, most people are not aware of its health benefits.

### 4.2. Consumer Acceptability of Biscuits Supplemented with Sorghum–Insect Meal

Sensory properties are important criteria accompanying the consumption of edible insects [41]. In the current study, it was thought that the sensory properties of the termites experienced by the consumers previously had contributed significantly to the termites being the most preferred. Therefore, we thought that a meal processed from termites would likely be the most acceptable in biscuits relative to meals of other insect types. The change in the color of biscuits supplemented with the sorghum–insect meal varied due to the use of insects in varying proportions. Taste was also a determining factor for the acceptance of biscuits supplemented with the sorghum–insect meal as those containing 5% and 10% insect concentration were more accepted and rated higher than the control. The derived smoky taste of the biscuits could also have been a contributing factor and could be attributed to the release of pheromones occurring at the surface of the insect organism, which are mainly responsible for taste and flavor [42]. The 100% wheat biscuits (control) had a smoother surface than those containing the sorghum–insect meal. Biscuits supplemented with the sorghum–insect meal were uneven at the surface. However, this observation did not seem to have compromised the product quality or acceptability, as the ratings were similar. The rough characteristics of biscuits supplemented with the sorghum–insect meal may be due to the hard, corneous endosperm cells of sorghum grain that remain intact during milling [43]. Sorghum starch granules that are encapsulated by hydrophobic cross-linked kafirins after baking could also have contributed to the rough characteristics [44]. These granules do not absorb adequate water for expansion and are perceived as gritty [45]. Similar results have also been reported by researchers who reported on sorghum biscuits [46,47]. An assessment of overall acceptability estimates the sum influence of the main sensory attributes, including appearance and taste, of the product on its sensory acceptability. In the current study, it seems that color, appearance of the surface of the biscuit samples, and taste were major sensory attributes that contributed to the overall acceptability of the biscuits.

Overall, the study revealed that biscuits supplemented with the sorghum–insect meal were more acceptable than the control biscuits. In addition, biscuit sample, B20 (15% sorghum and 5% insect substitution level) was more acceptable than those with higher concentrations of insects (B40 and B60). This result agrees with previous research of authors [48], who developed wheat buns enriched with edible termites. The authors found that wheat buns were more acceptable at 5% concentration of termites when compared to 20% concentration of termites. In a similar study, sensory analysis showed that the incorporation of termites in biscuits was acceptable up to 25% substitution [49]. Previous authors have also reported the high acceptability of sorghum-based biscuits [46,49,50]. These are promising findings especially where a lack of acceptance has often limited the idea of using insects as a food source [22].

### 4.3. Focus Group Discussion

Results presented in Table 5 shows that the perceptions of insects as food could be explained by the different levels of individual experience with eating insects, as prior experience is known to play an important role in how products are perceived [51,52]. Through the discussions, the influence of cultural exposure and individual experiences on insect consumption were revealed. The focus group discussions showed that consumer decisions behind the acceptance and rejection of insects as food are multifaceted. Consumers tended to draw different inferences according to their familiarity with insects. High levels of experience with eating a product results in inferences that predict the actual sensory properties more accurately, and low levels of experience result in less relevant inferences [53,54]. When participants were asked about their motivation for consuming insects, the taste of insects was a particularly important motivation amongst the consumers who reflected mostly on their eating experiences and gave reasons that related to their taste and how they enjoyed them (Table 5). Some participants preferred insects to be incorporated into a savory food product rather than a sweet product such as biscuit. This suggests that the food norms which consumers are accustomed to, are likely to play an important role in the acceptance of insects as a food source. According to Martins and Pliner [55], when prior experience is absent, the willingness to try new food products is often dependent on the level of interest and disgust, rather than on the expected experience of the sensory properties.

When asked about the benefits of insect consumption, most participants highlighted that it is about the taste pleasure rather than the additional benefits. The majority of the participants were not aware of the potential nutritional benefits of insects and once informed of these benefits, their interest and willingness increased. Therefore, more awareness is needed to enlighten people about the nutritional benefits associated with insect consumption. Consumers were also willing to accept insect as a food source if it were incorporated into other local food products. Hence, the incorporation of insects into other food products should be encouraged.

## 5. Conclusions

The findings of this study show that biscuits containing sorghum–insect meal were more acceptable to panelists than the control and this indicates that sorghum–insect meal could be successfully incorporated into food products. Sensory evaluation showed that biscuits supplemented with the sorghum–insect meal at 20% (15% sorghum and 5% insect substitution level) were acceptable to the panelists. Although, this study was carried out in a community where insects are widely consumed, the results may not be the case among non-insectivorous populations. Given the protein content of insects, especially termites used in this study, biscuits supplemented with the sorghum–insect meal could serve as an acceptable protein-rich, value-added food to improve the protein intake of those groups living in rural areas, where sorghum is a staple and insects are being consumed already, though not at nutritionally significant levels. This study revealed that more education needs to be conducted on the health and nutritional benefits of insect consumption, especially for those who live in rural areas. Focus group discussions revealed that people were more willing to consume insects if they were incorporated into their local foods. Hence, food product development aiming to increase utilization of insects as food should consider the cultural and individual expectations about the species of insect to be used as food and the food type that the consumers would prefer for incorporation of the insect.

## Figures and Tables

**Table 1:** Ratios of ingredients (sorghum, wheat, and insect) for biscuit formulation.

Ingredient	Relative Concentration (% w/w)			
Wheat flour	100	80	60	40
Sorghum meal	0	15	30	45
Insect meal	0	5	10	15
Identity of biscuit sample	control /B0	B20	B40	B60

Sorghum: insect meal (Ratio 3:1) replaced wheat flour at 0%, 20%, 40%, and 60% (w/w) levels.

**Table 2:** Total number and age of panelists who participated in sensory evaluation (*n* = 84).

Age Group (Years)	*n*	Male	Female
20–29	30 (35.7)	15 (17.9)	15 (17.9)
30–39	25 (29.8)	12 (14.3)	13 (15.5)
40–49	19 (22.6)	10 (11.9)	9 (10.7)
50–59	10 (11.9)	4 (4.8)	6 (7.1)

Figures in parentheses are percentages of the total sample (*n* = 84).

**Table 3:** Effect of the sorghum–insect meal on consumer acceptability of biscuits (*n* = 84).

Biscuit Samples	Color	Taste	Aroma	Texture	Overall Acceptability
B0	3.18 ^c^ ± 0.6	3.05 ^b^ ± 0.6	3.27 ^b^ ± 0.6	3.42 ^a^ ± 0.4	3.58 ^c^ ± 0.6
B20	3.42 ^a^ ± 0.6	3.21 ^a^ ± 0.5	3.46 ^a^ ± 0.6	3.45 ^a^ ± 0.5	4.23 ^a^ ± 0.6
B40	3.32 ^b^ ± 0.5	3.16 ^c^ ± 0.6	3.43 ^a^ ± 0.6	3.42 ^a^ ± 0.4	3.98 ^b^ ± 0.6
B60	3.17 ^c^ ± 0.7	2.96 ^d^ ± 0.5	3.04 ^c^ ± 0.6	3.45 ^a^ ± 0.4	3.77 ^c^ ± 0.6

Mean ± SD; Mean with different superscript letters (^a, b, c, d^) in the same column are significantly different (*p* < 0.05) according to the Least Significance Difference (LSD) test; B0: Control (100% wheat); B20: 15% sorghum and 5% insect substitution level; B40: 30% sorghum and 10% insect substitution level; B60: 45% sorghum and 15% insect substitution level.

**Table 4:** Demographic characteristics of panelists who participated in focus group discussions.

Age Group (Years)	*n*	Male	Female
20–29	5 (16.6)	3 (10)	6 (20)
30–39	9 (30.0)	1 (3.3)	4 (13.3)
40–49	11 (36.6)	3 (10)	10 (33.3)
50–59	5 (16.6)	2 (6.7)	1 (3.3)

Values in parentheses are percentages of total sample (*n* = 30).

**Table 5:** Consumer perceptions of insects as a food source.

THEMES	CONCEPTS	DISCUSSION	QUOTES
Cultural exposure and individual experience as determinants of acceptance	Sensory appeal	Participants possessed significant knowledge of how cooking influences the sensory properties because of cumulative experiences of preparing and consuming insects with their families. They expressed clear expectations of how insects should be prepared in order to achieve the best taste.	*“Each insect has a different taste, some insects have a savory taste, some insects have a bit of meat, some are soft and creamy.”* *“When they are fried in hot oil they become crispy which is different from boiled insects. If you boil insects, they lose their crunchiness.”*
Cultural exposure and individual experience as determinants of acceptance	Preparation methods	Participants agreed that they greatly enjoyed the taste of consuming whole insects as a snack. However, they were unsure if the same pleasure would be derived from consuming insects in the form of a food product as this would require milling the insects into a powder.	*“I eat insects because I use it as cooking ingredient for enhancing flavor. For example, vegetable soup with insects is more delicious than without insects.”* *“Insects can taste very nice when they are properly cooked and served in a way that’s appealing to the eyes.”*
Likelihood to purchase insect- based food products	Health benefits Environmental benefits	The participants showed more interest in buying insect-based foods when informed about the health benefits of consuming insects. Their interest was mainly motivated by the novelty of the experience and the purported environmental and health benefits. Their interest related to the sustainable and nutritious alternatives to meat.	*“The most important reason that I would eat insects is protein.”* *“I have quit eating meat because of my health so I think insect would serve as a good alternative.”* *“I am willing to consume any food that will keep me alive especially if it will be another source of income.”*
Will other familiar products improve the sensory-liking and willingness to buy insect-based foods?	Willingness to consume insects incorporated into other food products	Majority of the participants were willing to buy insect-based foods if there were various food products to choose from. Some participants expected insects to fit better with a savory food product, thereby the addition of insect to a sweet product such as biscuit was considered to be inappropriate. They further expressed that they would only consider adding insects to a food product of their choice, preferably local foods and in a way that does not take away from their eating pleasure.	*“Why should there be insects in biscuits anyway. It feels rather strange.”* *“I could try and use some insect-based ingredients or flour to prepare my own food and see if there’s a difference from the usual taste am used to.”*
